# Feasibility of assessing bone matrix and mineral properties *in vivo* by combined solid-state ^1^H and ^31^P MRI

**DOI:** 10.1371/journal.pone.0173995

**Published:** 2017-03-15

**Authors:** Xia Zhao, Hee Kwon Song, Alan C. Seifert, Cheng Li, Felix W. Wehrli

**Affiliations:** Laboratory for Structural, Physiologic and Functional Imaging, Department of Radiology, Perelman School of Medicine, University of Pennsylvania, 1 Founders Building, MRI Education Center, Philadelphia, PA, United States of America; Rensselaer Polytechnic Institute, UNITED STATES

## Abstract

**Purpose:**

To develop and evaluate an integrated imaging protocol for bone water and phosphorus quantification *in vivo* by solid-state ^1^H and ^31^P MRI.

**Materials and methods:**

All studies were HIPAA-compliant and were performed with institutional review board approval and written informed consent. Proton (^1^H) ultra-short echo-time (UTE) and phosphorus (^31^P) zero echo-time (ZTE) sequences were designed and implemented on a 3 T clinical MR scanner to quantify bone water and mineral *in vivo*. The left tibia of ten healthy subjects (including both genders, 49±15 y/o) was examined with a custom-built ^1^H/^31^P dual-frequency extremity RF coil. Total bone water (TW), water bound to the collagen matrix (BW) and bone ^31^P were quantified from MR images with respect to reference samples of known ^1^H or ^31^P concentration, and pore water (PW) was subsequently determined from TW and BW. Porosity index (PI) was calculated as the ratio between UTE images acquired at two echo times. MRI parameters were compared with bone density measures obtained by high-resolution peripheral quantitative CT (HR-pQCT).

**Results:**

The total scan time for the bone water and ^31^P quantification protocol was about 50 minutes. Average TW, BW, PW and ^31^P concentrations were 13.99±1.26, 10.39±0.80, 3.34±1.41 mol/L and 7.06±1.53 mol/L for the studied cohort, respectively, in good agreement with previous results conducted *ex vivo*. Average intra-subject coefficients of variation were 3.47%, 2.60% and 7.50% for TW, BW and PW and 5.60% for ^31^P. Negative correlations were observed between PW and vBMD (p<0.05) as well as between PI and ^31^P (p<0.05), while bone mineral content (BMC) estimated from ^31^P MRI and HR-pQCT were strongly positively correlated (p<0.0001).

**Conclusion:**

This work demonstrates the feasibility of quantifying bone water and mineral phosphorus in human subjects in a single MRI session with a clinically practical imaging protocol.

## Introduction

Cortical bone, which accounts for 80% of the skeleton by weight, consists of an organic substrate (also referred to as matrix) composed predominantly of type-I collagen, interspersed with mineral crystals of nonstoichiometric calcium apatite. Blood supply occurs through a system of interconnected pores (Haversian and Volkmann canals). In osteoporosis, an increasingly prevalent condition afflicting the older population [1, 2], thinning of the cortical shell occurs, along with pore expansion and depletion of mineral and matrix, thereby compromising the bone’s mechanical competence [3].

The current standard modality for osteoporotic fracture risk assessment is dual-energy X-ray absorptiometry (DXA), which measures gross density of bone material and, thus, cannot provide information on microstructure (such as cortical pore volume fraction and pore size distribution) or tissue mineralization. Consequently, DXA has low predictive accuracy—only 44% of non-vertebral fractures were found to occur in women with DXA-reported T-scores below -2.5 [4]. Similarly, DXA is unable to distinguish osteomalacia, a disorder in which bone is poorly mineralized but the pore volume fraction remains largely unchanged [5], from osteoporosis, where pore volume fraction increases but the remaining bone is normally mineralized.

Risk assessment has recently been augmented with the fracture risk assessment tool (FRAX) [6]. However, alternative techniques that can directly evaluate bone matrix, pore space, and mineralization *in vivo* are needed to provide more complete insight into the determinants of the bone’s mechanical competence.

Intracortical remodeling during aging, and more so in osteoporosis, involves expansion of pores [7]. Although most pores are beyond the resolution limit of *in vivo* imaging modalities, the portion of total bone water residing in the pore spaces (pore water, PW) scales inversely with matrix density [8] and has been shown to be quantifiable by MRI. On the other hand, although collagen protons themselves are not visible with clinical imaging equipment owing to their extremely short T_2_ (tens of microseconds), water bound to collagen (bound water, BW) scales linearly with bone matrix density. It has been shown that these two water pools can be distinguished from one another given their very different T_2_ [9] or T_2_* relaxation times [10]. *Ex vivo* studies have demonstrated quantification of pore and bound water based on MR images of human cortical bone [8, 11], as well as the correlation between these water pools and bone mechanical strength [12–14]. Techawiboonwong et al first quantified total bone water in the tibial cortex *in vivo* [15], while separation of the two water pools (BW and PW) *in vivo* has recently been reported by Manhard et al using T_2_-selective imaging sequences [16] and, more recently, by Chen et al using bi-exponential fitting of the T_2_* signal decay [17].

Quantitative MRI of ^31^P would potentially provide complementary insight into bone mineral properties. Animal studies have shown that ^31^P MRI-based quantification is able to detect impaired mineralization density in hypophosphatemia-induced osteomalacia in a rabbit model, as well as effects of anti-resorptive treatment in ovariectomized rats at 9.4 T [18–20]. The feasibility of *in vivo* ^31^P imaging in human subjects was shown first at 1.5 T [21] and, more recently at 3 T [22], albeit without quantification of ^31^P. Seifert et al measured phosphorus density by ^31^P solid-state MRI of human cortical bone of the tibia *ex vivo* at 7 T using custom-designed RF coil and pulse sequences [11]. However, there have so far been no reports on quantification of ^31^P density by *in vivo* human MRI.

The current work aims to demonstrate the feasibility of combined MRI-based *in vivo* bone water and mineral quantification, including discrimination of bound and pore water, as part of a single integrated imaging protocol.

## Materials and methods

### Human subjects

^31^P ZTE’s capability of detecting the ^31^P signal in the tibial shaft was initially evaluated on a 32-year old male in the presence of HA samples with ^31^P concentrations distributed in a wider range (3–7.5 mol/L). Subsequently, the entire protocol was executed in a cohort of healthy subjects. Inclusion criteria for enrollment were: 1) no medical history of diseases or treatments known to affect bone mineral homeostasis (e.g. mal-absorption syndromes, renal or hepatic disease, treatment with dexamethasone or methotrexate); 2) no conditions limiting normal physical activity (e.g. stroke, hip or leg fracture, rheumatoid arthritis); 3) body mass index < 30 kg/m^2^. All *in vivo* studies were done in compliance with HIPAA regulations, and were approved by the University of Pennsylvania’s IRB under protocol #823377. All subjects provided written informed consent. Ten healthy volunteers (two males, eight females, age range: 29 to 65 y/o, mean (SD) = 49 (15) y/o), recruited from the University of Pennsylvania (Philadelphia, PA), from July to August of 2016, participated in this study.

### Imaging protocol

MRI scans were performed on a 3 T TIM Trio system (Siemens Medical Solutions, Erlangen, Germany) using a custom-built transmit/receive ^1^H (123 MHz)-^31^P (49.9 MHz) dual-tuned birdcage coil (Rapid Biomedical, Rimpar, Germany). The complete protocol including localizer scans, ^31^P transmit power calibration and three radial imaging scans for quantification of bone water and phosphorus lasted approximately 50 minutes. The radial scans consisted of two proton (^1^H) ultra-short echo-time (UTE) sequences for quantification of total bone water (TW) and BW (from which PW was indirectly determined) and one phosphorus (^31^P) zero echo-time (ZTE) sequence for measuring bone phosphorus content. Details are given in the subsections below. All scans were carried out in the presence of a ^1^H density calibration sample (20% H_2_O/80% D_2_O, doped with 27 mmol/L of MnCl_2,_ corresponding to a H_2_O concentration of 11 mol/L, T_1_ = 4.3 ms, T_2_* = 320 μs) and two ^31^P density calibration samples (one consisting of a mixture of hydroxyapatite and calcium sulfate powders, [^31^P] = 7.5 mol/L, T_1_ = 46.2 s, T_2_* = 139 μs; and one consisting of pure hydroxyapatite powder, [^31^P] = 9.5 mol/L, T_1_ = 42.2 s, T_2_* = 145 μs), positioned in close proximity and directly anterior to the section of tibia being examined. This MRI procedure was applied in ten subjects, and was repeated an additional two times in a subset of three subjects to evaluate test-retest repeatability. Each of these three subjects dismounted the table and was repositioned between successive repetitions on the same day, or was scanned on a different day within a three-week period. MRI scans were centered at 38% tibia (38% of the tibia length from the medial malleolus), where the cortical bone is thickest [11]. High-resolution peripheral quantitative computed tomography (HR-pQCT) was also performed for comparison with ^31^P results.

#### ^1^H dual-echo UTE

**Fig 1:**
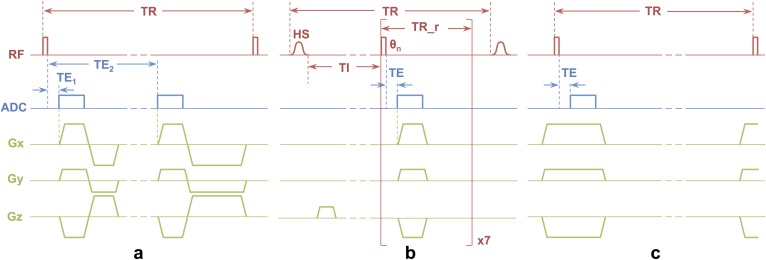
Diagrams of UTE and ZTE radial imaging sequences. a) ^1^H dual-echo UTE and b) IR_rUTE sequences for bone water quantification and porosity evaluation. In (a), two FIDs were collected following each excitation, at TE_1_ = 50 μs and TE_2_ = 4.6 ms. Spins were rewound after collecting the first FID but spoiled after the second. In (b), an HS pulse was applied to specifically invert long-T_2_ spins while saturating short-T_2_ spins, followed by an inversion time TI to null long-T_2_ spins, and 7 UTE readouts. c) ^31^P ZTE to quantify bone ^31^P concentration. Readout gradients are switched on prior to the RF pulse to ensure k-space is traversed with full speed following excitation.

TW was imaged using a 3D ^1^H dual-echo UTE sequence [23] (**Fig 1A**). An 80 μs rectangular radio frequency (RF) pulse was used for excitation, and two ‘echoes’, were sampled at 50 μs (TE_1_) and 4.6 ms (TE_2_) after the RF pulse. The prescribed field of view (FOV) was (250 mm)^3^, repetition time (TR) was 10 ms, and flip angle (FA) was 16°. Fifty thousand radial spokes, distributed to fully sample the spherical k-space, were collected at a dwell time of 4 μs in 8.3 min. The duration of the gradient ramp was 240 *μ*s, and 158 points were acquired along each radial spoke to populate an image matrix of 256x256x256. Images were reconstructed at an isotropic voxel size of (0.98 mm)^3^.

#### ^1^H IR-rUTE

BW was isolated and imaged using a 3D ^1^H inversion recovery-prepared rapid UTE (IR-rUTE) sequence [23, 24] (**Fig 1B**), which takes advantage of the differences in both T_1_ and T_2_ of bound and pore water. Due to its T_2_-selectivity, the adiabatic inversion pulse at the beginning of each TR inverts the long-T_2_ pore water magnetization while only saturating the short-T_2_ bound water spins [11]. After an appropriately chosen inversion recovery delay (TI), at which time the longitudinal magnetization of PW spins reaches zero while that of BW has recovered to a positive value, a series of seven spokes were acquired [24]. Thus only BW signal is detected within cortical bone. In the current implementation, adiabatic inversion was realized by a 5-ms, 5-kHz hyperbolic secant (HS) pulse, TI was set to 65 ms, and repetition time between consecutive inversions (TR) was 194 ms. Each of the seven UTE readouts was initiated by an 80 μs rectangular pulse, with flip angles increasing from 23° to 40° (variable flip angle scheme, VFA [24]) to create constant transverse magnetization from a diminishing reserve of longitudinal magnetization. The separation between each UTE readout was 2 ms. Twelve thousand spokes were collected at an effective TE of 50 μs resulting in 5.8 min total scan time. FOV, dwell time, number of points, and voxel size were the same as for dual-echo UTE.

#### ^31^P ZTE

Mineral phosphorus was imaged with the ZTE-PETRA technique [25] in lieu of the UTE strategy (**Fig 1C**). ZTE allows for faster k-space traversal, as no ramp sampling is involved. Therefore, given the extremely short T_2_* (<200 *μ*s [22]), ZTE has been found to have superior SNR relative to UTE under similar sequence parameter settings [26]. Although the first several ZTE readout points are lost during the transmit/receive switching time, the PETRA technique recovers the lost data within this sphere via single point imaging (SPI, all at the same effective TE)—mitigating point-spread function blurring of the image due to T_2_* exponential modulation of the k-space. ^31^P ZTE-PETRA was optimized for the experimentally observed relaxation parameters. TR and FA were 150 ms and 6.5°, rectangular RF pulse width was 16 μs, and 8000 spokes were sampled at a dwell time of 8 μs. PETRA radius (equal to the number of readout samples lost during transmit/receive switching time) was set to 6 (hence, the effective TE was dwell time × PETRA radius = 48 μs), for a total of 895 single points [25]. FOV was matched to that of the two ^1^H UTE sequences, but only 50 points along each spoke were acquired for reconstruction, leading to a reconstructed voxel size of (2.5 mm)^3^. Total scan time was 22.5 min. Since the scanner’s transmit power auto-calibration was not operational for nuclei other than ^1^H, the flip angle was calibrated for every subject prior to the ^31^P ZTE scan by incrementally stepping the power and maximizing the equilibrium calf muscle phosphocreatine signal.

#### HR-pQCT

HR-pQCT scans were performed on an XtremeCT II system (Scanco Medical, Brüttisellen, Switzerland). X-ray tube was operating at voltage/current of 68 kVp/1.47 mA. A 140×140×10.2 mm^3^ volume was imaged and reconstructed to a 2304×2304×168 matrix size, resulting in an isotropic spatial resolution of (61 μm)^3^. Only nine of the subjects received this scan because the scanner was unable to accommodate the limb size of one of the subjects.

### Quantification

Images were reconstructed by re-gridding to Cartesian k-space and standard Fourier transform [25]. Cortical water concentrations were determined from ^1^H images by referencing the signal within the tibial cortex to that of the MnCl_2_ doped calibration sample, and ^31^P concentrations were calculated similarly by referencing the tibial signal against that of the hydroxyapatite (HA) samples. Details on conversion of signal intensities to concentration are given in the following sections. Both image reconstruction and quantification were performed with custom programs written in MATLAB (Mathworks, Natick, USA).

#### Bone water quantification

TW was quantified from the central 45 slices (corresponding to a 44.1-mm thick slab) of the first echo image of the dual-echo UTE scan. Regions of interest (ROI) for cortical bone and calibration sample were drawn for each of these slices using a semi-automated segmentation algorithm [27]. The average intensity within the ROI of the calibration sample was used to compute the TW concentration within the cortical bone region on a pixel-by-pixel basis based on Eq (1):

$$\rho_{bone}=\rho_{ref}\frac{I_{bone}F_{ref}}{I_{ref}F_{bone}}e^{-TE(\frac{1}{T_{2\_ref}^{*}}-\frac{1}{T_{2\_bone}^{*}})}$$ (1)

where ρ_ref_ and ρ_bone_ are ^1^H densities in reference sample and cortical bone respectively, *I*_ref_ and *I*_bone_ are image intensities, while *F*_ref_ and *F*_bone_ represent the fractions of magnetization available for signal detection (see **Appendix**).

We note that even though T_1_ values of bone water and calibration samples differ, absolute concentrations can be computed from **Eq 1** since we know the chemical makeup, concentration and T_1_ of the calibration sample. The same arguments also apply to quantification of ^31^P mineral density below. BW maps were generated in the same way from the IR-rUTE images using the same ROIs based on **Eq 1**, but with *F*_ref_ and *F*_bone_ replaced by *IR_F*_*ref*_ and *IR_F*_*bone*_ respectively, which represent fractions of magnetization detected in IR-rUTE sequence (**Appendix**). Subtraction of the BW from the TW maps yielded a parametric image of PW. Median values were then used for statistical analysis. The following population average values for total and bound water relaxation times were used for bone water signal correction: T_1_total_ = 250 ms, T_2_total_* = 750 μs [27], T_1_bound_ = 145 ms, T_2_bound_* = 390 μs [28]. The ratio between the two images of dual-echo UTE sequence within the tibial cortex, referred to as porosity index (PI) [29], was also computed in a pixel-wise manner.

#### Bone phosphorus quantification

Due to the inherently lower SNR of ^31^P images, ^31^P concentration was quantified based on total signal within the volume of interest. To obtain the total ^31^P signal 18 consecutive axial images (corresponding to the same volume over which bone water was evaluated) were first complex-summed along the slice direction to produce an axial projection image with adequate SNR to clearly delineate the tibia and calibration sample boundaries. An ROI fully encompassing the tibia was then selected. Then a second complex summation was performed for pixels within this ROI, resulting in a single value whose magnitude represents total ^31^P signal. Signal from the reference samples was obtained in the same manner. Next, the actual volume of the tibial cortex was measured from the ROIs drawn for bone water quantification, whereas the volume of the HA sample was manually calculated using the inner diameter of the tubes. Volumetric average signal intensities of cortical bone and HA samples were subsequently determined, and after accounting for T_1_ and T_2_* differences, tibial ^31^P concentration was estimated via Eq (1). Population averages of T_1_ = 18 s and T_2_* = 160 μs were taken for bone ^31^P relaxation correction [22].

### Statistical analyses

Intra-subject coefficient of variation was calculated for all MRI-derived parameters for the three test-retest subjects. MRI-derived quantities were also compared with HR-pQCT reported volumetric bone mineral density (vBMD), and correlations among all parameters were determined. In addition, sub-region analysis was conducted by dividing the analysis slab into anterior, posterior, medial and lateral quadrants to determine possible regional differences in the measured parameters using analysis of variance (ANOVA), followed by post-hoc analysis. All statistical analyses were performed using JMP (SAS Institute Inc., Cary, USA).

## Results

**Fig 2:**
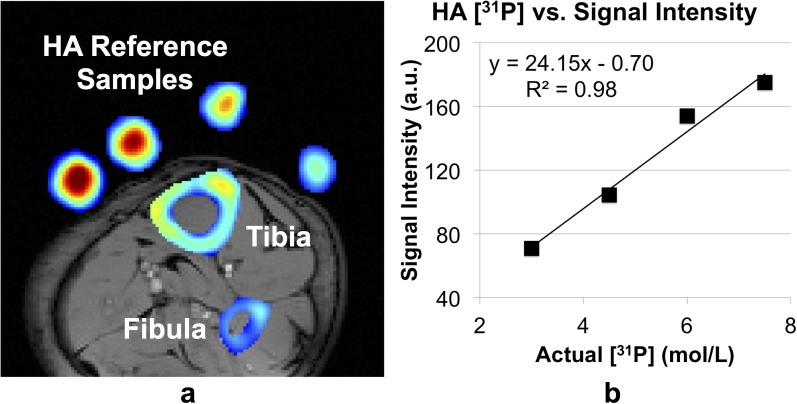
*In vivo* phosphorus intensity map and correlation with calibration samples. a) ^31^P image acquired in a 32 y/o male corresponding to a projection from 6-cm thick slab showing tibia, fibula, as well as four HA reference samples, superimposed on a proton anatomic image. b) Average signal intensity vs. ^31^P concentration for four HA reference samples. Sample concentrations were 3, 4.5, 6 and 7.5 mol/L respectively.

**Fig 2A** shows ^31^P signal intensities of the mid-tibial cortex superimposed on a ^1^H UTE image in one subject evaluated to determine feasibility of quantification along with HA reference samples. Average signal intensities of the four samples are plotted against their respective ^31^P concentrations in **Fig 2B**, showing the expected linear relationship between actual concentration and signal intensities (R^2^ = 0.98).

**Fig 3:**
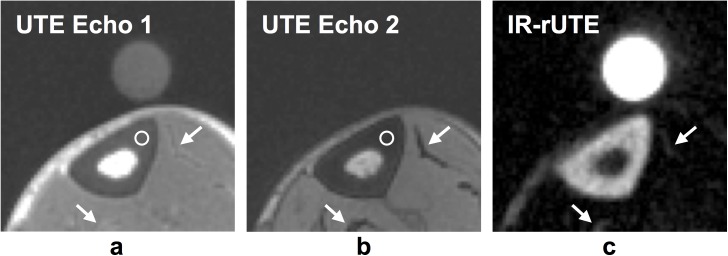
Total and bound water images of cortical bone. Bone water images from a 48 y/o male subject reconstructed from a) first and b) second echo of the dual-echo UTE sequence; c) BW image from the IR-rUTE sequence. Note that in (c), surrounding soft tissues as well as bone marrow within the medullary cavity is selectively suppressed via adiabatic inversion, leaving only short-T_2_^1^H density calibration sample and water tightly bound to collagen matrix. Image intensity measured from the circular ROI is noticeably higher in the first echo (0.32 versus 0.03 in the second echo). Also note similar intensity properties of the fasciae (arrows).

Representative dual-echo UTE and IR-rUTE images are displayed in **Fig 3A–3C**. In the two UTE images acquired at TE = 50 and 4600 μs the cortex appears dark with soft tissues slightly reduced in intensity at the longer echo time (**Fig 3A** and **3B**). However, the ROI (white circle) signal amplitude in the cortex is significantly greater for the first echo (0.32 versus 0.03). Also noticeable in **Fig 3B** are the fasciae (arrows), collagenous structures containing very short-T_2_ hydration water, appearing with background intensity as does the cortex. Further, the ^1^H reference sample present in the first echo image is no longer visible in the second echo image given the doped water’s very short T_2_* (~320 μs). In the inversion-recovery long-T_2_ suppressed image of the same slice (**Fig 3C**), muscle, subcutaneous and marrow fat are almost invisible while the cortex and fasciae now appear bright as does the reference sample. Average signal to noise ratio (SNR), here defined as the ratio of the mean signal amplitude within cortical bone to that of the background in magnitude images, measured across all subjects were 12 and 6 for the first and second-echo UTE images, respectively, and 17 in the IR-rUTE images.

**Fig 4:**
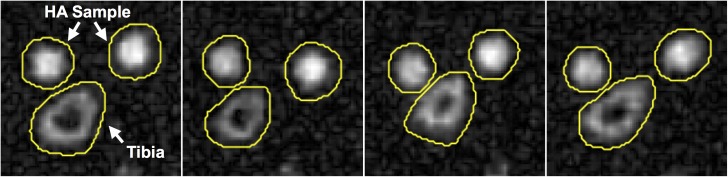
*In vivo* phosphorus images. ^31^P projection images (complex summation of a 45-mm thick slab) of four subjects with ROIs overlaid.

**Fig 5:**
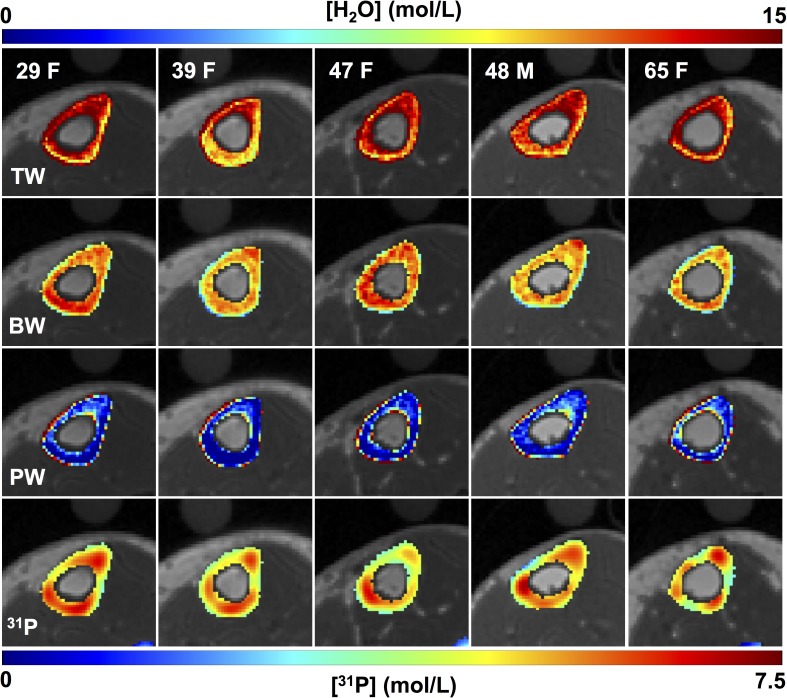
Bone water and phosphorus color maps in five of the ten study subjects. Differences in the distribution of both bone water and ^31^P are visually apparent across subjects. ^31^P maps were interpolated to match the resolution of the proton images.

**Fig 6:**
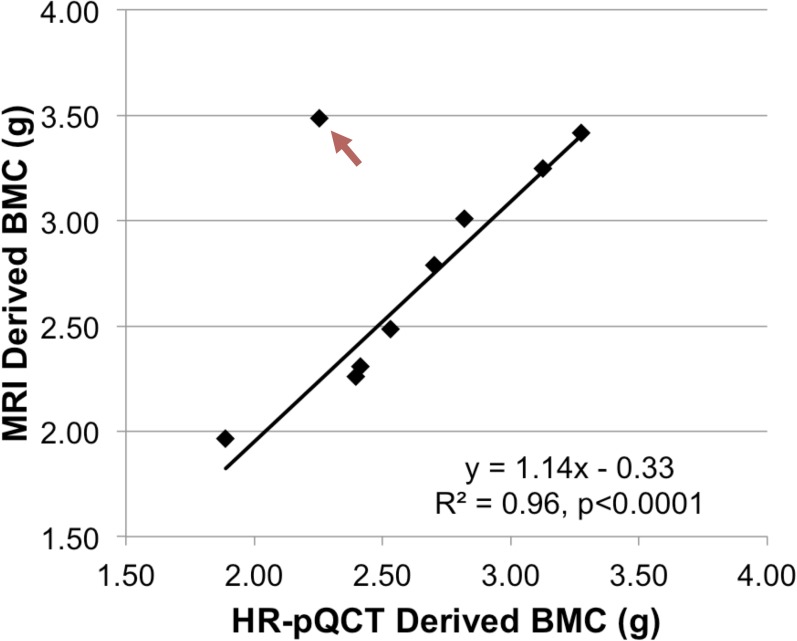
Comparison of BMCs estimated using MRI and HR-pQCT. BMC based on MRI-derived total ^31^P content plotted against HR-pQCT derived BMC for a 1-cm thick slab of tissue for nine subjects (only nine of the subjects could be scanned because the scanner was unable to accommodate the limb size of one subject). For HR-pQCT, BMC was computed by multiplying the reported vBMD with the volume of a 1-cm slab. The fitted line was obtained after excluding the 62 y/o female subject with abnormally high MRI-derived ^31^P concentration (red arrow).

**Fig 4** displays ^31^P projection images of four subjects (average SNR ~10 for tibia). The two HA reference samples and the tibia are unambiguously identified on this projection from a 45-mm thick volume, and the medullary cavity of the tibia is well delineated. **Fig 5** shows bone water and ^31^P density color maps for five of the study subjects. Bone mineral content (BMC) based on MRI-quantified total ^31^P content (assuming a bone apatite stoichiometry represented by Ca_5_(OH)(PO_4_)_3_) is plotted against that measured by HR-pQCT in **Fig 6**, showing the two quantities to be strongly correlated.

MRI-derived parameters and HR-pQCT reported vBMD are summarized in **Table 1** for all ten subjects. For the three subjects participating in test-retest validation, average values from three measurements are reported in the table. Average TW, BW and PW concentrations within this subject cohort were 13.99±1.26, 10.39±0.80 and 3.34±1.41 mol/L, respectively. The average PI was 0.34±0.06, and bone ^31^P concentration was 7.06±1.53 mol/L.

**Table 1:** Bone water, porosity and phosphorus quantification results for all ten subjects.

Subject	Bone Water (mol/L)			PI	^31^P (mol/L)	vBMD (mg/ccm)
(Gender/Age)	TW	BW	PW			
F/29	13.87	11.02	2.50	0.32	6.91	1016.8
F/31	13.19	10.75	2.15	0.37	6.34	1005.8
M/32	15.23	9.59	5.58	0.44	5.79	N.A.
F/39	11.71	10.15	1.39	0.33	6.49	1013.5
F/47	13.96	11.42	2.28	0.35	6.39	1023.0
M/48	13.77	10.96	2.55	0.26	7.03	1006.6
F/62	12.62	8.87	3.45	0.24	11.22	1023.0
F/63	15.54	9.94	5.40	0.36	7.48	986.9
F/65	14.44	10.06	4.07	0.37	6.30	994.1
F/65	15.54	11.16	4.06	0.31	6.62	1000.2
**Mean**	13.99	10.39	3.34	0.34	7.06	1007.77
**SD**	1.26	0.80	1.41	0.06	1.53	12.57

Sub-region analysis revealed significant inter-site differences for some but not all of the parameters extracted, including pore water and total water fraction (ANOVA, p<0.001). The significantly greater values for PW laterally suggest greater porosity at this location (**S1 Fig**).

A positive correlation was found between TW and PW (R = 0.81, p<0.005) while PW was negatively correlated with vBMD (R = -0.71, p<0.05), both expected. Both are plausible associations as a change in total water fraction is driven by the change in fractional pore space, and the latter is inversely related to osteoid volume fraction and thus, at constant mineralization, to BMD.

Test-retest results are given in **Table 2**. The CVs for two directly measured water quantities, TW and BW, are below 5% for all three subjects, but somewhat greater for PW (up to 8.5%), since instead of being a directly quantified parameter, the latter was obtained as the difference between two relatively large quantities, making it susceptible to errors in measurement. The average CV was 3.8% for the PI and 5.6% for bone ^31^P concentration.

**Table 2:** Test-retest repeatability in three subjects.

Subject	F/29					M/32					M/48				
(Gender/Age)															
Measurements	TW	BW	PW	PI	^31^P	TW	BW	PW	PI	^31^P	TW	BW	PW	PI	^31^P
**Scan 1**	14.16	11.24	2.59	0.33	6.55	15.35	9.61	5.71	0.43	5.82	13.05	10.42	2.34	0.26	7.09
**Scan 2**	14.08	11.10	2.63	0.34	7.39	15.56	9.69	5.87	0.43	6.08	14.19	11.20	2.77	0.26	7.37
**Scan 3**	13.36	10.73	2.29	0.30	6.78	14.77	9.48	5.16	0.45	5.48	14.07	11.27	2.55	0.27	6.63
**Mean**	13.87	11.02	2.50	0.32	6.91	15.23	9.59	5.58	0.44	5.79	13.77	10.96	2.55	0.26	7.03
**SD**	0.44	0.26	0.19	0.02	0.43	0.41	0.11	0.37	0.01	0.30	0.63	0.47	0.22	0.01	0.37
**CV (%)**	3.18	2.39	7.42	6.44	6.29	2.69	1.10	6.67	2.64	5.19	4.55	4.30	8.42	2.19	5.31

## Discussion and conclusion

By providing quantitative information on both bone water and ^31^P—surrogate markers of the organic and mineral phases of bone, respectively—the proposed dual-nucleus protocol has the potential to differentiate osteoporosis from demineralizing disorders such as osteomalacia, which is not achievable with currently available non-invasive modalities. As the respective surrogate marker of bone mineral content and matrix, the ratio of ^31^P and BW densities is expected to provide an indirect measure of the degree of mineralization. Because the two disorders differ in their underlying mechanisms, accurate diagnosis is crucial for effective medical intervention. Absence of ionizing radiation in MRI also makes it suitable for longitudinal studies involving one or more follow-up exams to monitor patient response to treatment (e.g. monitoring the response to anti-resorptive treatment for osteoporosis, or vitamin-D supplementation in osteomalacia).

Compared to bone water, ^31^P in bone is considerably more challenging to image due to the nucleus’ substantially shorter T_2_* and much longer T_1_ (160 μs and 18 s for ^31^P, respectively at 3 T). The rapidly decaying signal results in broadened point spread function (full width at half maximum ~4 mm at the gradient strength of 30mT/m used), potentially posing a hard limit on the maximally achievable spatial resolution. This limitation, however, is somewhat mitigated by the ZTE-PETRA sequence due to its more rapid traversal of k-space due to the absence of ramp-sampling, and constant TE within the central k-space (PETRA) region. The very short signal lifetime and long T_1_ of ^31^P also entail reduced SNR compared to that of ^1^H imaging. ^31^P also exists in lower concentration than ^1^H (~7 versus ~28 mol/L of ^1^H in bone water), and has a lower gyromagnetic ratio (by a factor of 2.5 relative to protons). The convergence of these factors renders *in vivo* phosphorus imaging with adequate resolution and SNR is extremely challenging. In fact, in order to achieve ^31^P SNR per unit time comparable to that of bone water, Wehrli et al projected that the spatial resolution would have to be relaxed by a factor of 20 [30].

In perhaps the first feasibility study of *in vivo* solid-state bone ^31^P MRI by Robson et al in the human tibia at 1.5 T [21], a 2D UTE sequence using a half-sinc pulse was used, requiring 64 averages to achieve sufficient SNR. The approximately 14-minute scan time, while clinically practical, yielded only a single slice of 60 mm thickness. Although quantification was performed in a whole-volume fashion in the current study as well, the ultimate goal is to be able to evaluate regional variations of ^31^P density. More recently, in very elegant work, Wu et al demonstrated the feasibility of *in vivo* 3D ^31^P imaging of the human wrist at 3 T in 37 minutes [22], although no density quantification was performed. In their implementation, a ZTE-type sequence was used in combination with a custom wrist coil, which provides relatively high detection sensitivity due to its close proximity to the imaging volume. In the only MRI-based human bone ^31^P quantification study to date, Seifert et al [11] reported an average ^31^P concentration of 6.74 mol/L in tibia specimens, versus 7.06±1.53 mol/L observed in the current *in vivo* work.

In the present study we utilized a birdcage coil with an inner diameter of 17 cm. Although some sensitivity is lost due to its large dimensions, it allows sufficient space for placement of calibration samples while providing a relatively large homogeneous B_1_ region. Measurements in ^1^H images of the homogeneous Siemens doped water phantom (3.75 g NiSO_4_·6H_2_O + 5 g NaCl per 1000 g of H_2_O) confirmed that signal variation was less than 10% within the volume of interest used in this study. Although the ^31^P transmit B_1_ was not measured directly, it was assumed to have similar homogeneity as that generated by the concentric ^1^H coil.

MRI based bone water quantification at the mid-tibia has been reported in several prior studies. Techawiboonwong et al reported an average TW concentration of 17.4% and 28.7% by volume (corresponding to 9.7 and 15.9 mol/L) in pre- and postmenopausal women, respectively [15]. More recently, Manhard et al reported an average BW concentration of 13.9 mol/L and PW concentration of 3.7 mol/L in an *in vivo* study of a small group of subjects [16]. TW, BW and PW concentrations of 13.99±1.26, 10.39±0.80 and 3.34±1.41 mol/L found in the current study are consistent with these earlier findings. Moreover, our test-retest experiments demonstrated repeatability for TW, BW and ^31^P measurements *in vivo* on the order of 5–8%, therefore rendering the method well-suited for longitudinal studies.

Exclusion of an extreme outlier (see **Fig 6**) suggests negative correlations between vBMD and age (R = -0.71), and between BW and PI (R = -0.65) as well as a positive correlation between BW and vBMD (R = 0.67) although these associations did not quite reach statistical significance (p<0.08), likely due to limited power of this feasibility study.

There was no significant correlation between MRI-derived ^31^P concentration and HR-pQCT derived vBMD in contrast to a previous *ex vivo* study in human cortical samples using similar methodology [11] reporting a positive correlation between the MRI-derived ^31^P concentration and CT derived BMD (R = 0.68, P<0.005). The same study also reported a positive correlation between BW and ^31^P concentration (R = 0.77, P<0.005). However, the current study did yield a strong positive correlation between BMC measured with MRI and that measured with HR-pQCT (R = 0.98, p<0.0001), as shown in **Fig 6**. Although this correlation was obtained with the same outlier exclusion stated above, statistical significance was still present even without exclusion of this particular subject (R = 0.67, p<0.05).

The rationale behind choosing the tibia as the imaging location is based on the following considerations. First, its proximity to the body surface and the overall geometry of the lower leg makes the tibia technically more amenable to examine with an optimized RF coil than more deep-lying structures such as femoral neck. Second, the mid-tibial cortex is relatively thick (5–7 mm). Third, since at stance, for instance, a large proportion of stress that governs remodeling in bipeds is along the vertical axis of the body, it is plausible that bone loss at the tibia is similar to that at typical fracture sites such as the proximal femur and spine, but this conjecture obviously would require detailed scrutiny in future studies. Fourth, degenerative bone disease, notably osteoporosis, is a systemic disorder. It has long been known that age-related cortical bone pore volume expansion occurs at multiple anatomic locations, with detailed studies having been conducted at the humerus [31], the femoral shaft [32], radius [33], or ilium [34]. We also note that in prior work by some of the authors, MRI measures of the calcaneus, for instance, distinguished osteoporotic fracture patients from controls as well as or better than did BMD of the proximal femur or vertebrae [35]. Sub-region analysis showed site-specific differences for some of the parameters. While the clinical significance of such observations is currently not known and beyond the scope of this article, it is likely that remodeling-related effects in response to drug intervention are anatomic site-dependent.

In this work, UTE was used for imaging bone water and ZTE for bone phosphorus. Seifert et al showed experimentally that ZTE-PETRA provides SNR superior to its UTE counterpart for ^31^P imaging [26]. Theoretically, bone water imaging should also benefit from ZTE for similar reasons. However, the peak B_1_ of the coil limited the maximally achievable flip angle of a 16 μs rectangular pulse for ^1^H to about 9°. In order to increase the flip angle (and SNR), pulse duration would have to be increased. This is not possible in ZTE: a pulse duration greater than twice the dwell time would cause severe excitation selectivity within the imaging field-of-view [36]. Therefore, UTE was utilized in lieu of ZTE for ^1^H imaging so the flip angle could be optimized by increasing the RF pulse duration.

The present work has limitations. As a feasibility study it falls short of providing adequate power to test some of the associations examined. Second, the study used a ‘population average’ value of cortical bone T_1_ values in computing the MR parameters. However, Seifert et al demonstrated that the longitudinal relaxation time of bone ^31^P scales with the degree of mineralization [26], thus the assumed T_1_ may potentially deviate from actual values thereby adversely impacting quantification. This is less of a problem as long as the bone is normally mineralized (unlike in bone demineralizing disorders such as osteomalacia). The errors incurred from using an average ^31^P T_1_ value need be examined, as well as the feasibility of T_1_ measurement within a maximum allowable procedure time of one hour. While deriving BW concentration from IR-rUTE images, BW magnetization was assumed to recover from zero after each inversion since the residual longitudinal magnetization immediately following the HS pulse has been found to be negligible [37]. Nevertheless, it is conceivable that saturation is not always complete. Further, a single inversion delay was used to null all long-T_2_ species, as it has been shown that the optimal delay is similar for tissue water and fat as long as TR<T_1_ [23]. However, **Fig 3C** lends strong support of effective suppression of both bone marrow and surrounding soft tissues. Another potential source of systematic error is imperfect nulling of PW spins at the time of the excitation pulse given the wide range of their T_1_ values [14].

In conclusion, the present feasibility study, while limited in scope, highlights the potential of solid-state MRI for the quantitative evaluation of cortical bone matrix and mineral properties in the form of an integrated, single-session quantitative study of bone water and mineral phosphorus.

## Appendix

### ^1^H UTE (^31^P ZTE)

The transverse magnetization immediately after RF pulse in UTE and ZTE is given as:

$$F=M_{xy}=f_{xy}∙\frac{1-e^{\frac{-TR}{T_{1}}}}{1-f_{z}∙e^{\frac{-TR}{T_{1}}}},$$ (A1)

where f_xy_ and f_z_ are mapping functions reflecting the response of longitudinal and transverse magnetization to a rectangle pulse the duration of which (τ) is comparable to the spin’s effective transverse relaxation time (T_2_*), and are defined as:

$$f_{xy}=\gamma B_{1}\tau e^{\frac{-\tau }{2T_{2}^{*}}}sinc(\sqrt{{\left(\gamma B_{1}\tau \right)}^{2}-{\left(\frac{\tau }{2T_{2}^{*}}\right)}^{2}}),$$ (A2)

and

$$f_{z}=e^{\frac{-\tau }{2T_{2}^{*}}}[\cos\sqrt{{\left(\gamma B_{1}\tau \right)}^{2}-{\left(\frac{\tau }{2T_{2}^{*}}\right)}^{2}}+\frac{\tau }{2T_{2}^{*}}sinc(\sqrt{{\left(\gamma B_{1}\tau \right)}^{2}-{\left(\frac{\tau }{2T_{2}^{*}}\right)}^{2}})]$$ (A3)

In the regime of τ << than T_2_*, these two mapping functions reduce to sin(γB_1_τ) and cos(γB_1_τ) respectively.

### ^1^H IR-rUTE

The signal of inversion recovery-prepared UTE is however, of a different steady-state. In order to derive an analytical solution, the protons of bound water and reference sample were assumed to be fully saturated immediately after each adiabatic inversion (actually, numeric Bloch equation simulation indicated a residue of only ~5%), therefore, longitudinal magnetization of bound water (reference sample) right before the first UTE readout is:

$$M_{z}^{-}(1)=1-e^{-\frac{TI}{T_{1}}},$$ (A4)

with TI being the inversion recovery delay. And the longitudinal and transverse magnetization right after the RF pulse could be written as:

$$M_{xy}(1)=f_{xy}∙M_{z}^{-}(1),$$ (A5)

$$M_{Z}^{+}(1)=f_{z}∙M_{z}^{-}(1),$$ (A6)

where f_xy_ and f_z_ are the same mapping function as described above. By the time of next RF excitation, transverse magnetization would completely vanish while longitudinal magnetization would have recovered for a period of (TR_r- τ) (**Fig 1B**), so the initial M_z_ for all the rest six UTE readouts would be:

$$M_{z}^{-}(i)=1-[1-M_{z}^{+}(i-1)]e^{-\frac{\left(TR\_r-\tau \right)}{T_{1}}},$$ (A7)

and the average value of transverse magnetization following all seven excitations is used to represent steady-state signal for bound water and reference sample:

$$IR\_F=\frac{\sum_{i=1}^{7}M_{xy}(i)}{7}$$ (A8)

## Supporting information

S1 Fig Regional dependence of PW. In order to investigate potential spatial dependence of MRI-derived parameters, the tibial cortex of each subject was divided into four quadrants: anterior, posterior, medial and lateral. ANOVA was applied to compare each parameter among these spatial locations, and PW was found to be significantly higher in the lateral region than in the other three quadrants.
(PNG)
