# Correction: Feasibility of assessing bone matrix and mineral properties *in vivo* by combined solid-state ^1^H and ^31^P MRI

**DOI:** 10.1371/journal.pone.0192186

**Published:** 2018-01-29

**Authors:** Xia Zhao, Hee Kwon Song, Alan C. Seifert, Cheng Li, Felix W. Wehrli

The authors note that the volume used in computing the phosphorus concentration of the calibration samples was incorrect, which subsequently affected the computed in vivo phosphorus concentrations.

In Fig 5, the range of the color bar for [^31^P] (mol/L) is incorrect. Please see the corrected Fig 5 here.

**Fig 5:**
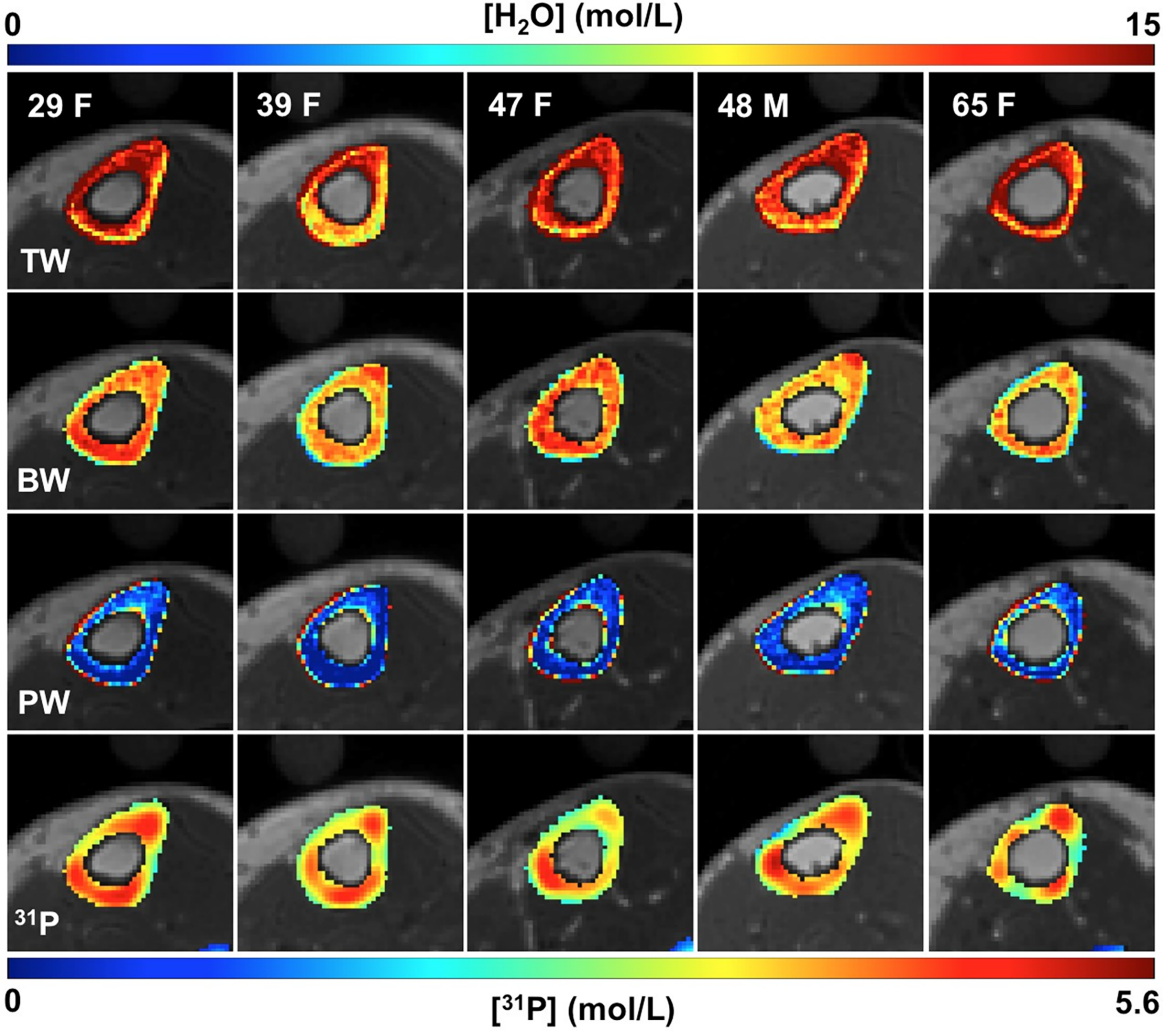
Bone water and phosphorus color maps in five of the ten study subjects. Differences in the distribution of both bone water and ^31^P are visually apparent across subjects. ^31^P maps were interpolated to match the resolution of the proton images.

In Fig 6, the bone phosphorus concentration values are incorrect. Please see the corrected Fig 6 here.

**Fig 6:**
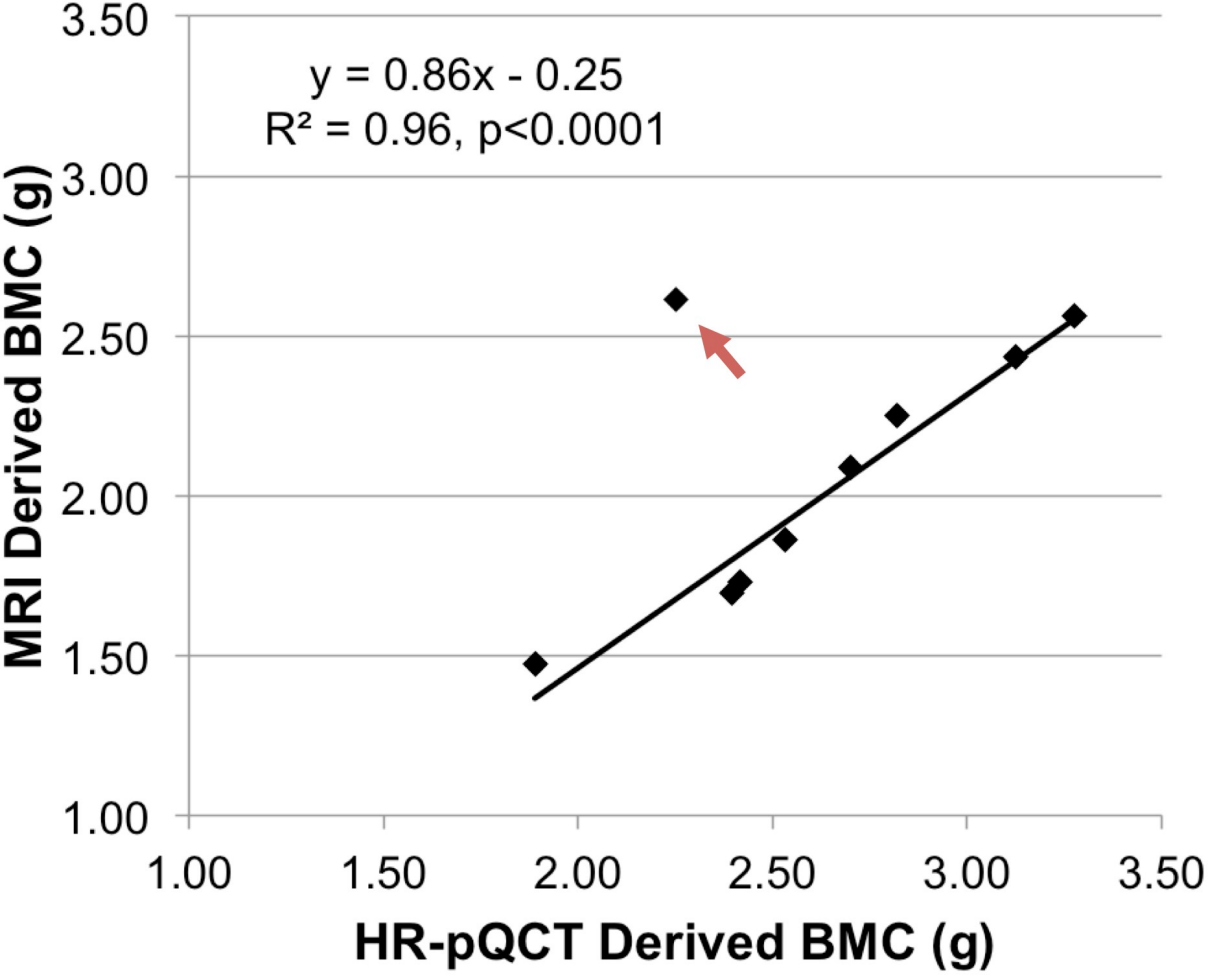
Comparison of BMCs estimated using MRI and HR-pQCT. BMC based on MRI-derived total ^31^P content plotted against HR-pQCT derived BMC for a 1-cm thick slab of tissue for nine subjects (only nine of the subjects could be scanned because the scanner was unable to accommodate the limb size of one subject). For HR-pQCT, BMC was computed by multiplying the reported vBMD with the volume of a 1-cm slab. The fitted line was obtained after excluding the 62 y/o female subject with abnormally high MRI-derived ^31^P concentration (red arrow).

In Table 1, the values in the column “^31^P (mol/L)” are incorrect. Please see the corrected Table 1 here.

**Table 1:** Bone water, porosity and phosphorus quantification results for all ten subjects.

Subject (Gender/Age)	Bone Water (mol/L)			PI	^31^P (mol/L)	vBMD (mg/ccm)
	TW	BW	PW			
F/29	13.87	11.02	2.50	0.32	5.18	1016.8
F/31	13.19	10.75	2.15	0.37	4.75	1005.8
M/32	15.23	9.59	5.58	0.44	4.35	N.A.
F/39	11.71	10.15	1.39	0.33	4.86	1013.5
F/47	13.96	11.42	2.28	0.35	4.79	1023.0
M/48	13.77	10.96	2.55	0.26	5.27	1006.6
F/62	12.62	8.87	3.45	0.24	8.41	1023.0
F/63	15.54	9.94	5.40	0.36	5.61	986.9
F/65	14.44	10.06	4.07	0.37	4.72	994.1
F/65	15.54	11.16	4.06	0.31	4.96	1000.2
**Mean**	13.99	10.39	3.34	0.34	5.29	1007.77
**SD**	1.26	0.80	1.41	0.06	1.15	12.57

In Table 2, the “^31^P” Measurements in the “F/29”, “M/32”, and “M/48” sections are incorrect. Please see the corrected Table 2 here.

**Table 2:** Test-retest repeatability in three subjects.

Subject (Gender/Age)	F/29					M/32					M/48				
Measurements	TW	BW	PW	PI	^31^P	TW	BW	PW	PI	^31^P	TW	BW	PW	PI	^31^P
**Scan 1**	14.16	11.24	2.59	0.33	4.91	15.35	9.61	5.71	0.43	4.37	13.05	10.42	2.34	0.26	5.32
**Scan 2**	14.08	11.10	2.63	0.34	5.55	15.56	9.69	5.87	0.43	4.56	14.19	11.20	2.77	0.26	5.53
**Scan 3**	13.36	10.73	2.29	0.30	5.08	14.77	9.48	5.16	0.45	4.11	14.07	11.27	2.55	0.27	4.97
**Mean**	13.87	11.02	2.50	0.32	5.18	15.23	9.59	5.58	0.44	4.35	13.77	10.96	2.55	0.26	5.27
**SD**	0.44	0.26	0.19	0.02	0.33	0.41	0.11	0.37	0.01	0.23	0.63	0.47	0.22	0.01	0.28
**CV (%)**	3.18	2.39	7.42	6.44	6.40	2.69	1.10	6.67	2.64	5.20	4.55	4.30	8.42	2.19	5.36
