# Supplementary figures and images for: Feasibility of assessing bone matrix and mineral properties in vivo by combined solid-state 1H and 31P MRI
Source: PLoS One. 2017 Mar 15;12(3):e0173995. doi: 10.1371/journal.pone.0173995 (PMC5352014; doi:10.1371/journal.pone.0173995)

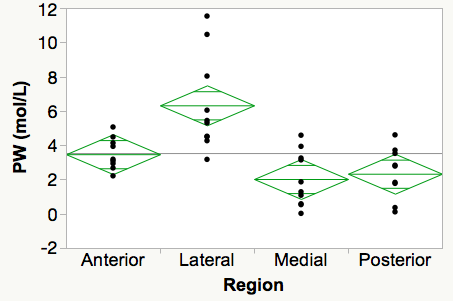

Supplement: S1 Fig — In order to investigate potential spatial dependence of MRI-derived parameters, the tibial cortex of each subject was divided into four quadrants: anterior, posterior, medial and lateral. ANOVA was applied to compare each parameter among these spatial locations, and PW was found to be significantly higher in the lateral region than in the other three quadrants. (PNG) [file pone.0173995.s001.png]
